# Effects of Warming and Clipping on Ecosystem Carbon Fluxes across Two Hydrologically Contrasting Years in an Alpine Meadow of the Qinghai-Tibet Plateau

**DOI:** 10.1371/journal.pone.0109319

**Published:** 2014-10-07

**Authors:** Fei Peng, Quangang You, Manhou Xu, Jian Guo, Tao Wang, Xian Xue

**Affiliations:** Key Laboratory of Desert and Desertification, Chinese Academy of Sciences, Cold and Arid Regions Environmental and Engineering Research Institute, Chinese Academy of Sciences, Lanzhou, China; DOE Pacific Northwest National Laboratory, United States of America

## Abstract

Responses of ecosystem carbon (C) fluxes to human disturbance and climatic warming will affect terrestrial ecosystem C storage and feedback to climate change. We conducted a manipulative experiment to investigate the effects of warming and clipping on soil respiration (Rs), ecosystem respiration (ER), net ecosystem exchange (NEE) and gross ecosystem production (GEP) in an alpine meadow in a permafrost region during two hydrologically contrasting years (2012, with 29.9% higher precipitation than the long-term mean, and 2013, with 18.9% lower precipitation than the long-tem mean). Our results showed that GEP was higher than ER, leading to a net C sink (measured by NEE) over the two growing seasons. Warming significantly stimulated ecosystem C fluxes in 2012 but did not significantly affect these fluxes in 2013. On average, the warming-induced increase in GEP (1.49 µ mol m^−2^s^−1^) was higher than in ER (0.80 µ mol m^−2^s^−1^), resulting in an increase in NEE (0.70 µ mol m^−2^s^−1^). Clipping and its interaction with warming had no significant effects on C fluxes, whereas clipping significantly reduced aboveground biomass (AGB) by 51.5 g m^−2^ in 2013. These results suggest the response of C fluxes to warming and clipping depends on hydrological variations. In the wet year, the warming treatment caused a reduction in water, but increases in soil temperature and AGB contributed to the positive response of ecosystem C fluxes to warming. In the dry year, the reduction in soil moisture, caused by warming, and the reduction in AGB, caused by clipping, were compensated by higher soil temperatures in warmed plots. Our findings highlight the importance of changes in soil moisture in mediating the responses of ecosystem C fluxes to climate warming in an alpine meadow ecosystem.

## Introduction

Global mean temperature has increased by 0.76°C since the year 1850 and is predicted to rise an additional 1.8–4°C by the end of the 21^st^ century [1]. Elevated global temperature can substantially impact the global carbon (C) budget, resulting in positive or negative feedbacks to global climate change [2], [3]. The balance between C fixed by photosynthesis and C emitted to the atmosphere through plant and heterotrophic respiration determines the rate of terrestrial C storage [4].

Studies have shown that global warming could stimulate both ecosystem C uptake and emission across various terrestrial biomes [5]. However, the response of net C balance to warming is highly variable because of different temperature and soil moisture sensitivities in the processes that control C uptake and emission [6]. It is generally assumed that the terrestrial ecosystem might act as a net C source under a global warming scenario because the processes controlling ecosystem C emission are more sensitive to higher temperatures than the processes controlling C uptake [3], [7], [8]. However, some evidence indicates that warming could increase net C uptake, and global C models project enhanced terrestrial CO_2_ uptake in response to warming through the middle of this century [9], [10]. Current and completed experimental studies that have investigated warming effects have focused mostly on net primary productivity (NPP), biomass and soil respiration [5], [11], from which the change in the C balance change was estimated. However, responses of gross primary production (GPP) and ecosystem respiration (ER), the major components net ecosystem exchange (NEE), to warming in field experiments [12] have received less attention in the alpine area [13].

Mowing (clipping) or grazing in grasslands, which account for 20% of the land use of the global terrestrial ice-free surface, may have substantial effects on ecosystem C fluxes, especially on a short-term basis [14]. Clipping would result in rapid changes in nutrient cycling [15], vegetation cover, plant community composition [16], and soil microclimate [17]. Collectively, these processes appear to stimulate the rate of ecosystem C cycling, however, their impacts on the net C balance are inconsistent [18], [19].

Carbon stored in permafrost at high latitudes and in mountain areas is one of the major components of the terrestrial C pool. It is estimated that soils in the permafrost regions store as much as 1672 Pg C (1 Pg = 10^15^ g), which is equivalent to double the atmospheric C pool [20], [21]. Ecosystems in permafrost regions are C sinks because microbial decomposition of soil organic matter is inhibited under low annual mean temperature, and there is limited availability of organic C in frozen soil [22]–[24]. Altered growing season length, and changes in plant growth, ecosystem energy exchange and land use, together with the thawing of permafrost under a changing climate are projected to enhance the capability of ecosystem C uptake [21]. However, these altered dynamics do not appear to be able to compensate for the C released from thawing permafrost, resulting in ecosystems in permafrost regions acting as positive feedback to global change [21].

The Qinghai-Tibet Plateau (QTP) is experiencing a “much greater than average” increase in surface temperature, based on data observed at meteorological stations [25] and predictions from coupled climate-carbon models [1]. Grassland in the QTP is the largest vegetation unit of the Eurasian continent and covers an area of approximately 2.5 million km^2^ [26]. Grazing is the most prevalent land use practice in the grassland. Results from eddy covariance measurements showed that the alpine meadow in the QTP is a weak C sink with annual variations [23], [24]. Several studies have examined responses of ER and aboveground biomass to warming and clipping [17], [27], [28], in which the alpine meadow is thought to be a net C sink based on C balance calculations [28]. However, no field experiment has been conducted in the permafrost region of the QTP to measure the response of NEE, which provides a direct measure of the C balance. We conducted a two-year warming and clipping experiment to investigate how NEE and its components (GPP and ER) respond to warming and clipping, and how the associated changes in soil moisture, soil temperature, above- and belowground biomass affect the responses of ecosystem C fluxes in the permafrost region of the QTP.

## Materials and Methods

### Experimental site

The study site is situated in the region of the Yangtze River source, inland of the QTP near the Beilu River research station (34°49′N, 92°56′E, no specific permissions were required for activities in this location) at an altitude of 4635 m. This area has a typical alpine climate: mean annual temperature is −3.8°C and monthly air temperature ranges from −27.9°C in January to 19.2°C in July. Mean annual precipitation is 290.9 mm, of which over 95% falls during the warm growing season (May to October). Mean annual potential evaporation is 1316.9 mm, mean annual relative humidity is 57%, and mean annual wind velocity is 4.1 m s^−1^ [29]. The study site is a winter-grazed range, dominated by alpine meadow vegetation: *Kobresia capillifolia*, *K. pygmaea*, and *Carex moorcroftii*, with a mean plant height of 5 cm. Plant roots occur mainly within the 0–20 cm soil layer, and average soil organic C is 1.5%. The soil development is weak, and the soil belongs to alpine meadow soil (Chinese soil taxonomy), or is classified as a Cryosol according to World Reference Base, with a Mattic Epipedon at a depth of approximately 0–10 cm, and an organic-rich layer at a depth of 20–30 cm [30]. The parent soil material is of fluvioglacial origin and is composed of 99% sand. The Mattic Epipedon lowers the saturated soil water content, but increases soil water storage, and plant roots are dense and compressed within this layer. Permafrost thickness observed near the experimental site is 60–200 m and the depth of the active layer is 2.0–3.2 m [29], [31]. However, because of climatic warming, the thickness of the active layer has been increasing at a rate of 3.1 cm y^−1^ since 1995 [32]. The experimental field is on a mountain slope with a mean incline of 5°. Detailed information about the soil properties is presented in Table 1.

**Table 1:** Results (F-values) of a three-way ANOVA on the effects of warming (W), clipping (C), measuring month (M), and their interactions on soil respiration (Rs), ecosystem respiration (ER), net ecosystem exchange (NEE) and gross ecosystem production (GEP).

	M	W	C	M×W	M×C	W×C	M×C×W
**Rs**	88.3**	3.9∧	0.1	0.1	0.3	4.2*	1.1
**ER**	21.8**	8.3**	0.2	0.4	0.1	0.1	0.4
**NEE**	43.0**	4.8**	0.8	1.2	0.4	0.1	0.1
**GEP**	44.3**	9.5**	0.0	0.7	0.1	0.1	0.1
**Rs/ER**	1.5	0.3	0.1	2.5**	1.8	4.0**	0.1
**ER/GEP**	13.3**	0.4	0.5	2.1∧	0.9	2.1	0.8
**AGB**	117.0**	3.8∧	22.2**	1.2	2.3∧	0.3	2.1∧
**RB**	1.0	1.3	2.0	0.1	0.1	3.0∧	0.2
**AGB/RB**	26.6**	10.3**	19.6**	6.6**	8.1**	13.0**	8.5**

Significance: ∧, *P*<0.1; *, *P*<0.05; **, *P*<0.01.

### Experimental design and measurement protocols

#### Experimental design

A two factorial experimental design (warming and clipping) was used with five replicates in each of the four treatments, i.e. unclipped control (UC), clipped control (CC), unclipped warming (UW) and clipped warming (CW). In total, 20 plots (2×2 m) were used in a complete randomized block distribution in the field. Plots were selected for homogeneity of topography, soil texture, aboveground biomass, and species composition. In each warmed plot, one 165 cm×15 cm infrared heater (MR-2420, Kalglo Electronics Inc., Bethlehem, PA, USA) was suspended in the middle of each plot at a height of 1.5 m above the ground with a radiation output of 150 watts m^−2^. The heating has been operating continuously since July 1^st^ 2010. To simulate the shading effects of the heaters, one “dummy” heater, made of a metal sheet with the same shape and size as the heaters, was also installed in the control plot.

Plants in the clipped plots were clipped at the soil surface on an annual basis, usually in last September. The rotational grazing system is that two ranches for each family, one is for the summer grazing and another is for the winter grazing. The rotational use of ranches is implemented usually in September. We consulted to the owner and they ensured that grassland of our study site is a winter grazing ranch. In our study, the clipping treatment was conducted in last September. One sheep needs 1.46×10^6^ g grass per year [33] and the carrying capacity for alpine meadow is 1.39 head of sheep per hectare. Based on those data, about 203 g m^−2^ grass per year would be grazed for the alpine meadow. The aboveground biomass in September 2013 was about 320 g m^−2^. In the clipping treatment, biomass cut was <320 g m^−2^. Therefore we believe that our clipping treatment provided a reasonable simulation of local grazing practices.

#### Measurement protocol

Air temperature, water vapor pressure and relative humidity were monitored automatically at a height of 20 cm above the soil surface in the center of each plot using a Model HMP45C probe (Campbell Scientific Inc., Bethlehem, PA, USA). Nine thermistors were installed to monitor soil temperatures at depths of 5, 15, 30, 60, 100, 150, 200, 250 and 300 cm. All the probes were connected to a CR1000 datalogger (Campbell Scientific Inc.). Data recorded every 10 min were averaged and reported as daily values. Pavelka *et al.* (2007) stated that for grassland ecosystems, surface soil temperature is the most suitable depth for measuring soil temperature because of the optimized regression coefficient between surface soil temperature and soil respiration [34]. Therefore, we used soil temperature measured at a depth of 5 cm in the following analyses.

An EnviroSmart sensor (Sentek Pty Ltd., Stepney, Australia), which used frequency domain reflection, was used to monitor volumetric soil moisture at depths of 0–10, 10–20, 20–40, 40–60 and 60–100 cm. These soil moisture data were also recorded using a CR1000 datalogger. When analyzing the relationships between C fluxes and soil moisture, we used the daily average soil moisture data that were collected when ecosystem C flux measurements were conducted.

Soil respiration (Rs) was measured by using Licor-6400-09 (Lincoln, NE, USA) on PVC collars 5 cm in height and 10.5 cm in diameter, which were permanently inserted 2–3 cm into the soil in the center of each plot. Small living plants were cut at the soil surface at least one day before measurements to eliminate the effect of respiration from aboveground biomass [35]. ER and NEE were measured with a transparent chamber (0.5×0.5×0.5 m) attached to an infrared gas analyzer (IRGA, Licor-6400, Lincoln, NE, USA). The transparent chamber is a custom-designed chamber made of Polytetrafluoroethene (4 mm in thickness) with light transmittance about 99%. During measurements, a foam gasket was placed the chamber and the soil surface to minimize leaks. One small fan ran continuously to mix the air inside the chamber during measurements. Nine consecutive recordings of CO_2_ concentration were taken in each plot at 10 s intervals during a 90 s period. Following the measurement of NEE, the chamber was vented for several minutes and covered with an opaque cloth for measuring ER, as the opaque cloth eliminated light (and hence photosynthesis). CO_2_ flux rates were determined from the time-course of the CO_2_ concentrations used to calculate NEE and ER. The method used was similar to that reported by Steduto *et al*. (2002) [36] and Niu *et al*. (2008) [37]. Gross ecosystem productivity (GEP) was the calculated as the sum of NEE and ER. Rs, NEE and ER were measured in each plot on a monthly basis from May to September in 2012 and 2013.

Aboveground biomass (AGB) was obtained from a step-wise linear regression with AGB as the dependent variable, and coverage and plant height as independent variables. 100 small plots (30 cm×30 cm) were included in the regression analysis (AGB = 22.76×plant height +308.26×coverage −121.80, R^2^ = 0.74, *P*<0.01). Coverage of each experimental plot was measured using a 10 cm×10 cm frame in four diagonally divided subplots replicated eight times. Plant height was measured 40 times by a ruler and averaged for each experimental plot. A biomass index was used as the ratio of the derived biomass on any given date to the maximum biomass during the entire study period [38]. Root biomass (RB) was obtained from soil samples that were air-dried for one week and passed through a 2- mm diameter sieve to remove large particles. Roots were separated from the soil by washing, and a 0.25-mm diameter sieve was used to retrieve fine roots. Living roots were separated from dead roots by their color and consistency [39]. Separated roots were dried at 75°C for 48 h.

### Data analysis

Temperature and soil moisture data used in analyses were from January 1^st^ 2012 to July 18^th^ 2013 because a power failure prevented data from being collected from July 19^th^ 2013 onwards. The effect of the warming and clipping treatments on soil temperature (5 cm), soil moisture (0–10 cm), Rs, ER, NEE, GEP, AGB and RB were determined with a three-way analysis of variance (ANOVA) using SPSS Version 18.0. (SPSS, Inc., Chicago, IL, USA).

Relationships between C fluxes and soil microclimate (soil temperature and soil moisture) were examined using daily soil microclimate data that were collected when ecosystem C fluxes were measured. Linear regression analyses were used to examine the relationships of C fluxes with abiotic (soil moisture and soil temperature) and biotic factors (monthly AGB and RB).

## Results

### Microclimate

**Figure 1:**
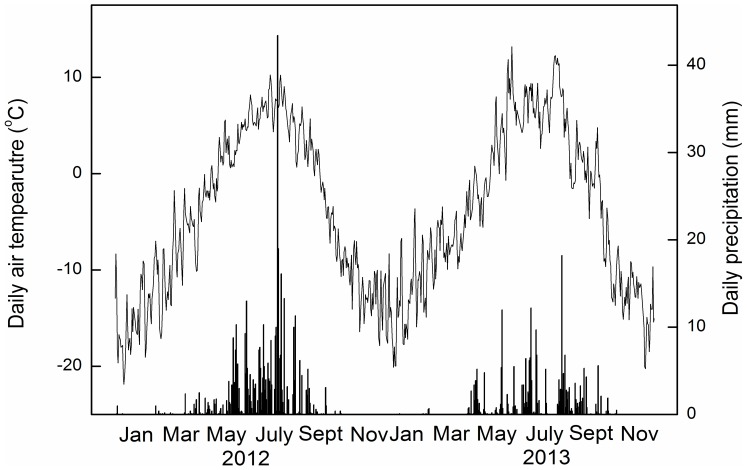
Daily precipitation (columns) and daily mean air temperature (line) in 2012 and 2013. Data are from the micro-meteorological station adjacent (approx. 100 m) to the experimental plots.

In comparison to the long-term average (1981–2008) mean annual air temperature (MAT, −5.1°C), higher MAT values were recorded in 2012 and 2013 (−3.5°C and −3.8°C, respectively). Annual precipitation in 2012 (420.1 mm, Fig. 1) was higher than the long-term mean annual precipitation (294.5 mm), but it was lower than the long-term mean in 2013 (238.8 mm) (Fig. 1).

**Figure 2:**
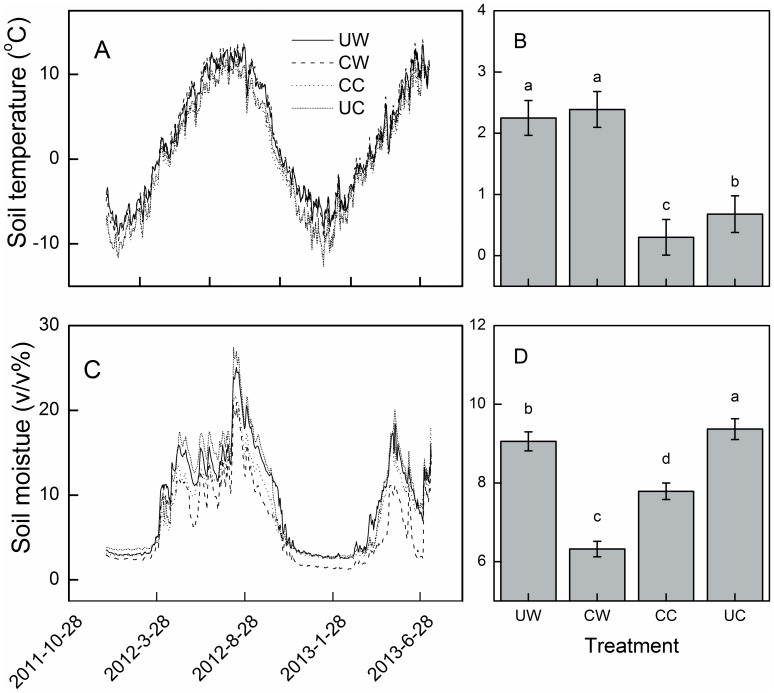
Daily soil temperature measured at a depth of 5 cm and volumetric soil moisture measured at a depth of 0–10 cm (A, C), and effects of warming and clipping on average (2012–2013) soil temperature (B) and soil moisture (d). UW, unclipped warming; CW, clipped warming; CC, clipped control; UC, unclipped control.

Experimental warming significantly elevated annual mean soil temperature (Figs. 2A and 2B, *P*<0.05). In unwarmed plots, the average daily soil temperature at 5 cm depth was 0.65°C and 0.14°C in 2012 and 2013, respectively. Warming significantly increased the soil temperature by 1.96°C (2012) and 1.59°C (2013) (Fig. 2B). The average daily soil temperature in unclipped plots was 1.69°C and 0.99°C in 2012 and 2013, respectively, but was unaffected by clipping. Volumetric soil moisture measured over 0–10 cm fluctuated greatly over the study period (Fig. 2C). The average daily soil moisture in unwarmed plots was 9.31 (v/v%) and 7.22 (v/v%), and warming significantly reduced soil moisture by 7.4% and 17.4% (*P*<0.05) in 2012 and 2013, respectively (Fig. 2D). The average daily soil moisture in unclipped plots was 10.08 (v/v%) and 7.61 (v/v%), and clipping significantly decreased it by 28.3% and 36.5% in 2012 and 2013, respectively (Fig. 2D).

### Warming and clipping effects on C fluxes

**Figure 3:**
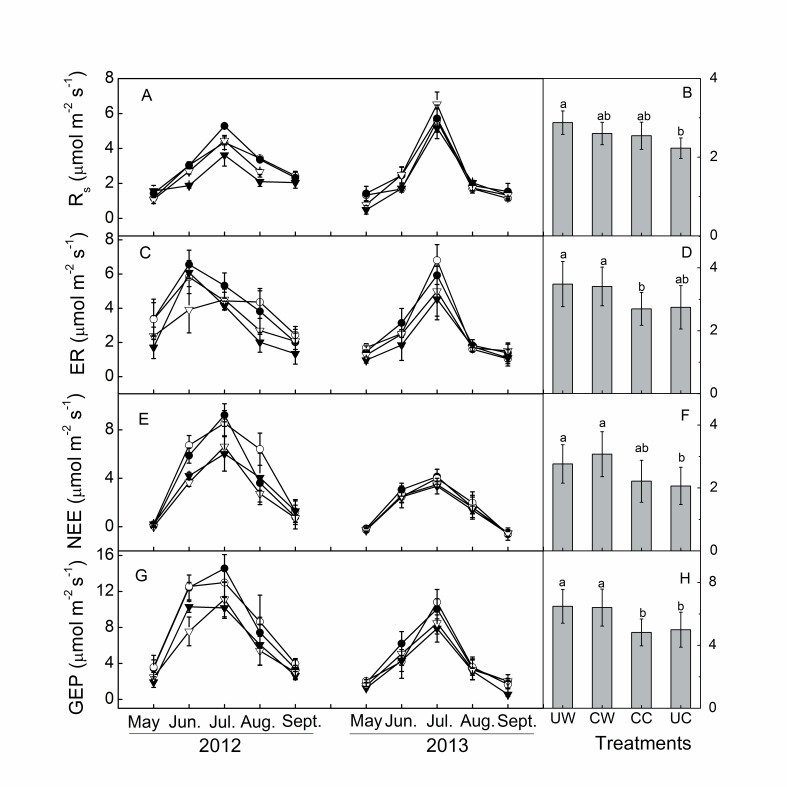
Mean monthly and overall means of soil respiration (Rs, A, B), ecosystem respiration (ER, C, D), net ecosystem exchange (NEE, E, F) and gross ecosystem production (GEP, G, H). Clipping was conducted in September 2011 and 2012. UW, unclipped warming; CW, clipped warming; CC, clipped control; UC, unclipped control.

**Figure 4:**
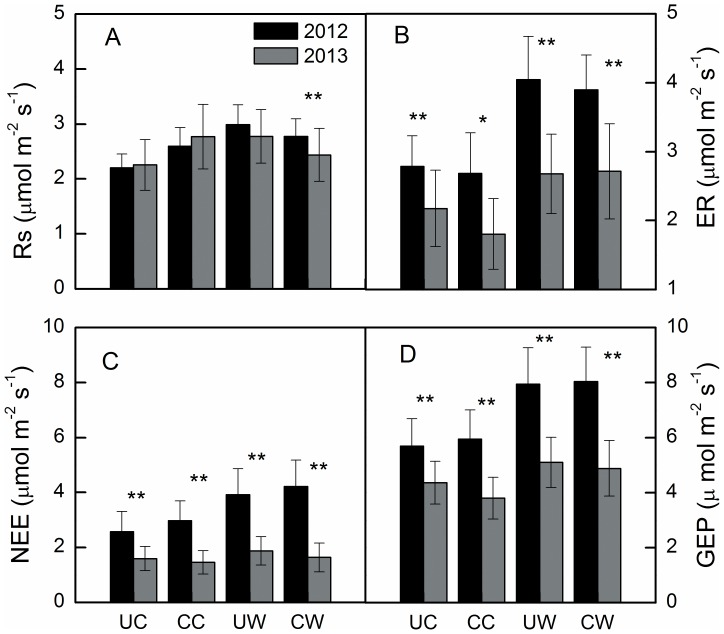
Mean annual soil respiration (Rs), ecosystem respiration (ER), net ecosystem exchange (NEE) and gross ecosystem production (GEP) under unclipped control (UC), clipped control (CC), unclipped warming (UW) and clipped warming (WC) treatments in 2012 and 2013. Symbols above the bars represent significant differences at p<0.05 (*) and p<0.01 (**).

The temporal dynamics of Rs, ER, NEE, and GEP followed the seasonal patterns of air and soil temperature in both years, which peaked in mid-growing season (Figs. 1 and 3). Substantial inter-annual variations in ecosystem C fluxes were observed in this study (Fig. 3). The annual average ER, NEE and GEP were all significantly higher in 2012 than in 2013 (Fig. 4) in all treatments, but higher Rs in 2012 was only observed in the CW treatment (Fig. 4A). On average, NEE, ER and GEP were 47%, 22% and 34% higher, respectively, in 2012 than in 2013.

Warming significantly increased NEE (*P* = 0.03), whereas no significant effects of clipping (*P* = 0.37) or its interaction with warming (*P* = 0.83) were detected (Table 1). When analyzed separately by year using a three-way ANOVA, warming only significantly increased NEE in 2012, by 28.5% (Table 2). Warming induced an enhanced growing season mean NEE in 2012, which was lower in clipped (17%) than in unclipped plots (30%). Measuring date had a significant effect on NEE, but the interaction of measuring date with warming or clipping had no effect on NEE in either year (Table 2).

**Table 2:** Results (F-values) of a three-way ANOVA on the effects of warming (W), clipping (C), measuring month (M), and their interactions on soil respiration (Rs), ecosystem respiration (ER), net ecosystem exchange (NEE) and gross ecosystem production (GEP) in contrasting years.

	M	W	C	M×W	M×C	W×C	M×C×W
**2012**							
**Rs**	54.2**	11.6**	0.8	2.2∧	0.3	5.1*	1.4
**ER**	13.9**	9.4**	0.1	0.6	0.4	0.01	1.2
**NEE**	46.9**	8.6**	2.6	2.0	1.3	0.0	0.04
**GEP**	40.3**	12.2**	0.2	0.9	0.6	0.1	0.2
**ER/GEP**	16.6**	0.05	0.2	0.6	0.1	0.8	0.8
**AGB**	33.9**	2.1	2.0	0.6	0.4	0.5	0.2
**RB**	7.2**	0.7	1.0	0.5	0.09	2.8	0.2
**AGB/RB**	2.4∧	1.2	2.2	0.5	0.2	1.6	0.2
**2013**							
**Rs**	78.3**	0.1	0.02	0.8	0.6	3.3∧	0.6
**ER**	31.0**	2.6	0.2	1.1	0.2	0.2	0.3
**NEE**	51.2**	1.1	0.3	0.3	0.1	0.01	0.1
**GEP**	44.2**	3.2∧	0.9	0.7	0.4	0.2	0.3
**ER/GEP**	22.1**	9.0**	11.0**	13.9**	8.3**	8.8**	10.9**
**AGB**	89.9**	0.06	20.1**	1.3	0.8	0.5	1.1
**RB**	2.9*	0.7	1.2	0.2	0.3	0.8	0.2
**AGB/RB**	20.7**	1.5	6.2*	1.5	2.3∧	2.6	2.7*

Significance: ∧, *P*<0.1; *, *P*<0.05; **, *P*<0.01.

Similar to NEE, average GEP was significantly increased by warming (*P* = 0.003) but not by clipping (*P* = 0.97) or by their interaction (*P* = 0.87, Table 1). When analyzed separately by year using a three-way ANOVA, warming significantly increased average GEP (Table 2) by 2.13 µ mol m^−2^s^−1^ in 2012 and marginally enhanced it by 0.82 µ mol m^−2^s^−1^ in 2013. The increased GEP caused by warming was lower in clipped (23%) than in unclipped plots (28.3%) in 2012, but GEP was higher in clipped (22.6%) than in unclipped plots (13.4%) in 2013.

Warming also significantly increased average Rs (*P* = 0.052) and ER (*P* = 0.005), but clipping had no significant effect on average Rs (*P* = 0.73) or ER (*P* = 0.66, Table 1). Similar to NEE, when analyzed separately by year using a three-way ANOVA, the effect of warming on ER and Rs was only significant in 2012 (Table 2), which increased by 25.8% and 17%, respectively. The interaction between warming and clipping had a significant effect on Rs but not on ER (Table 1). The average increase in Rs caused by warming was lower in clipped (5.9%) than in unclipped plots (27.4%).

There was no significant effect of warming or clipping on the ER to GEP ratio (ER/GEP, *P* = 0.51 and *P* = 0.46 for 2012 and 2013, respectively, Table 1). When analyzed separately by year using a three-way ANOVA, ER/GEP was significantly affected by warming, clipping, measurement date and their interactions in 2013 (Table 2).

### Warming and clipping effects on biomass

**Figure 5:**
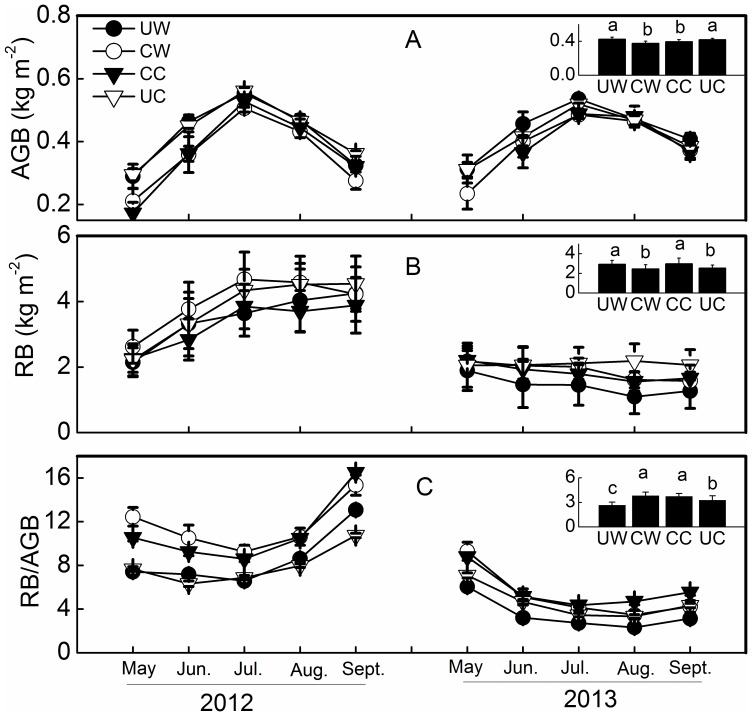
Temporal variations and overall means (inserted panels) of aboveground biomass (AGB, A), root biomass (RB, B) and the ratio of RB to AGB (RB/AGB, C). Clipping was conducted in September 2011 and 2012. See Figures 2 and 3 for notes and abbreviations.

Similar to the inter-annual variation of ecosystem C fluxes, RB was significantly lower in 2013 (by a value of 50.2%) than in 2012, whereas there was no difference in AGB over the two growing seasons (Fig. 5).

Warming marginally increased AGB (p = 0.053) and clipping significantly reduced AGB (p<0.001), but there was no significant effect on RB (p = 0.26 and p = 0.16 for warming and clipping, respectively, Table 1). When analyzed separately by year using a three-way ANOVA, warming had no significant effect on AGB or RB in either year, whereas clipping significantly reduced AGB in 2013 (Table 2). The reduction in AGB by clipping was higher in unwarmed (14.4%) plots than in warmed plots (10.3%) in 2013.

### Impacts of biotic and abiotic factors on ecosystem C fluxes

**Figure 6:**
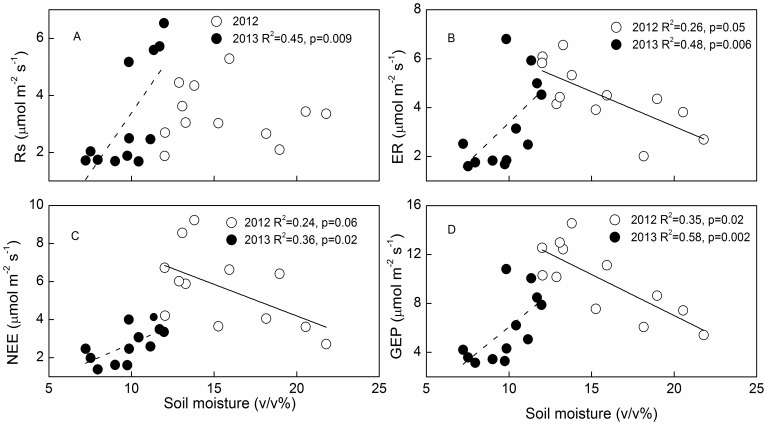
The relationships between soil moisture (0–10 cm) and ecosystem C fluxes: soil respiration (Rs), ecosystem respiration (ER), net ecosystem exchange (NEE) and gross ecosystem productivity (GEP) in 2012 (hollow circles) and 2013 (solid circles), respectively. Soil moisture and ecosystem C fluxes data were the average for all plots in each month. Data for both years were collected from June to August.

Over the two growing seasons, there was no clear relationship between ER, NEE, GEP and soil moisture, but there was a quadratic relationship between these variables and soil moisture when May and September data were excluded (Table 3). The optimal soil moisture for ER, NEE and GEP was about 15% (Table 3). When plotted separately, ER, NEE, and GEP decreased linearly with increasing soil moisture in 2012, and increased linearly with increasing soil moisture in 2013 (Fig. 6).

**Table 3:** Fitted quadratic models of the relationships between ecosystem respiration (ER), net ecosystem exchange (NEE), gross ecosystem production (GEP) and soil moisture (θ, v/v%, 10 cm). Max. F, θ represents the value of θ when ER, NEE and GEP are at their maximum.

	ER/µmol m^−2^s^−1^	NEE/µmol m^−2^s^−1^	GEP/µmol m^−2^s^−1^
Fitted model	ER = −0.058θ^2^+1.71θ–7.72	NEE = −0.076θ^2^+2.37θ-12.53	GEP = −0.139θ^2^+4.21θ-21.02
R^2^	0.31	0.45	0.36
p	0.007	<0.001	<0.001
Max. F, θ/%	14.7%	15.6%	15.1%

**Figure 7:**
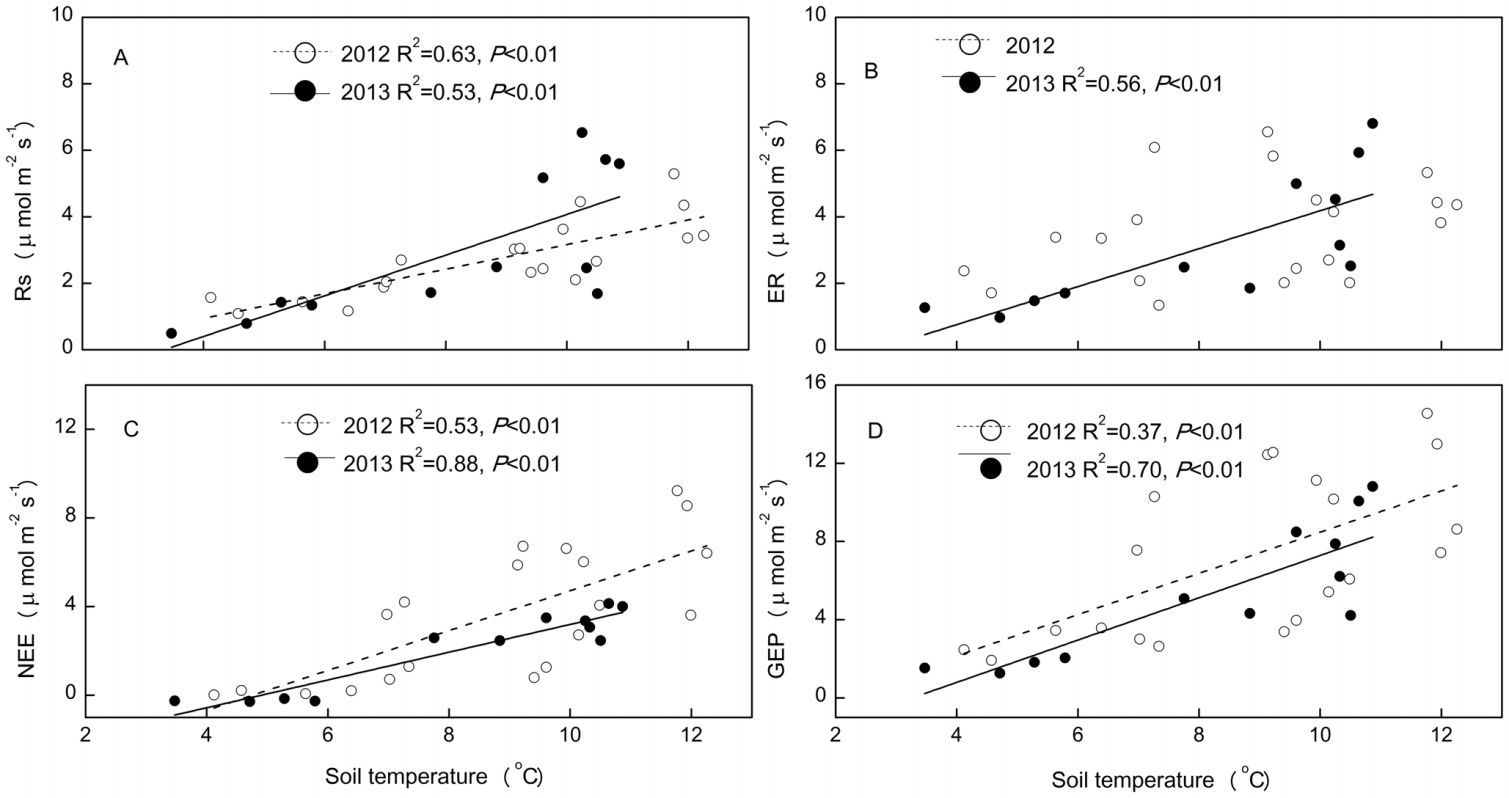
The relationships between soil temperature (5 cm) and ecosystem C fluxes: soil respiration (Rs), ecosystem respiration (ER), net ecosystem exchange (NEE) and gross ecosystem productivity (GEP). Soil temperature and ecosystem C fluxes data were the average of all plots in each month.

The temperature response curves for Rs, ER, NEE and GEP in 2012 were quite similar to those recorded in 2013. However, the slope of relationship between Rs and soil temperature was higher in 2012 than in 2013, but that between NEE and soil temperature was smaller in 2012 than in 2013 (Fig. 7).

**Figure 8:**
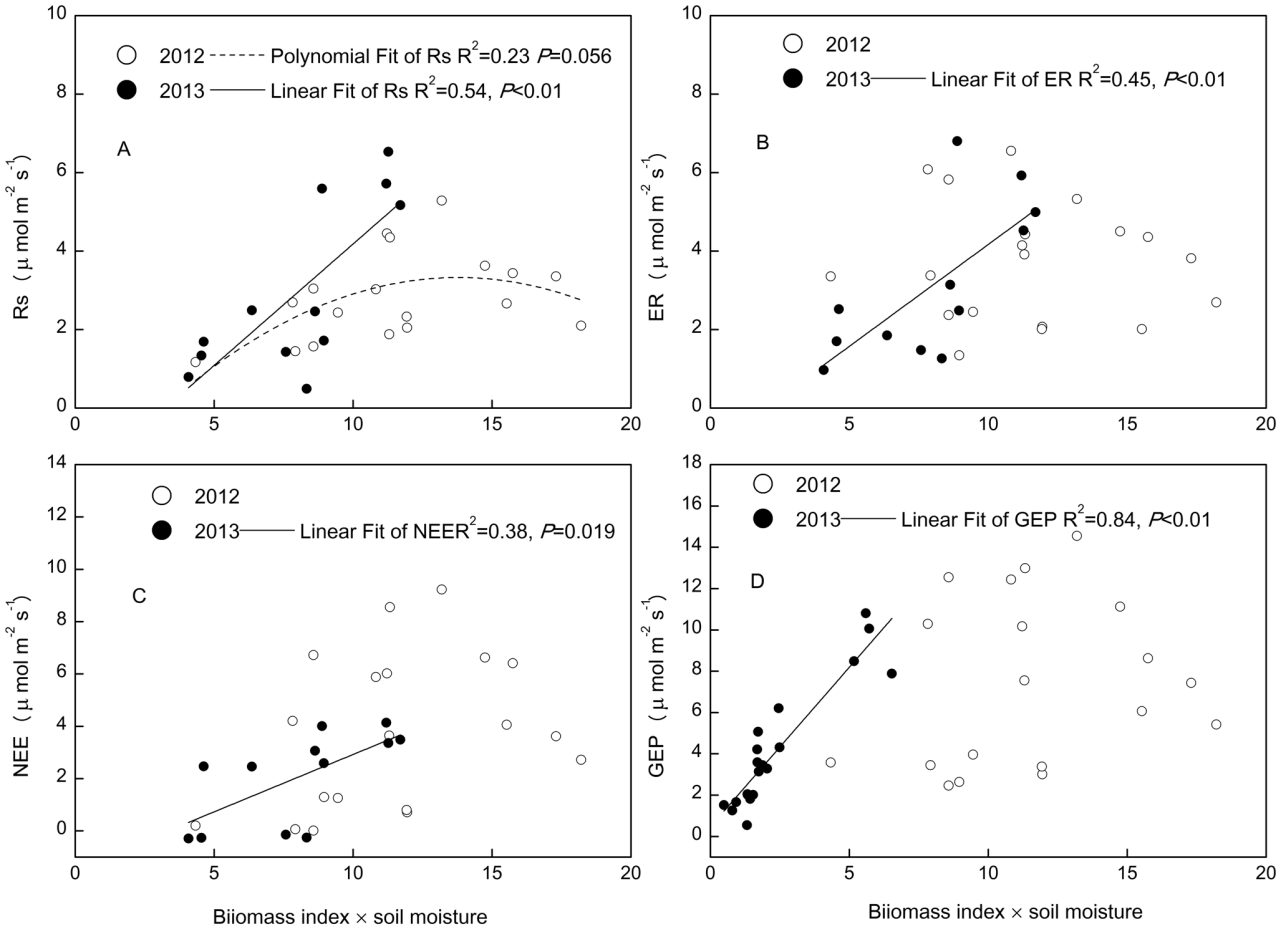
The relationships between changes in ecosystem C fluxes: soil respiration (Rs), ecosystem respiration (ER), net ecosystem exchange (NEE) and gross ecosystem productivity (GEP), and the changes in the product of abveground biomass index and soil moisture during 2012 and 2013.

**Figure 9:**
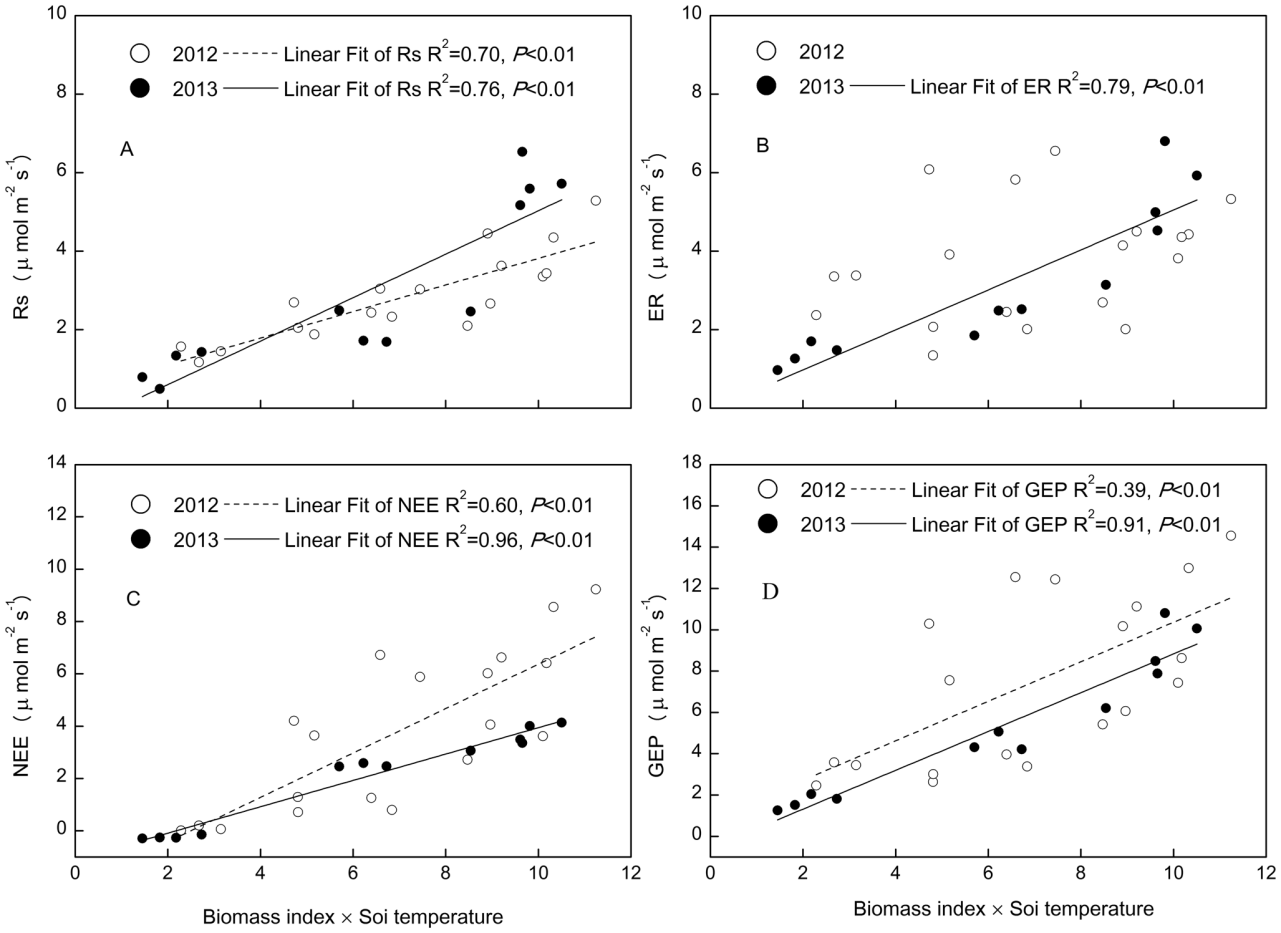
The relationships between changes in ecosystem C fluxes: soil respiration (Rs), ecosystem respiration (ER), net ecosystem exchange (NEE) and gross ecosystem productivity (GEP), and the changes in the product of aboveground biomass index and soil temperature during 2012 and 2013.

**Figure 10:**
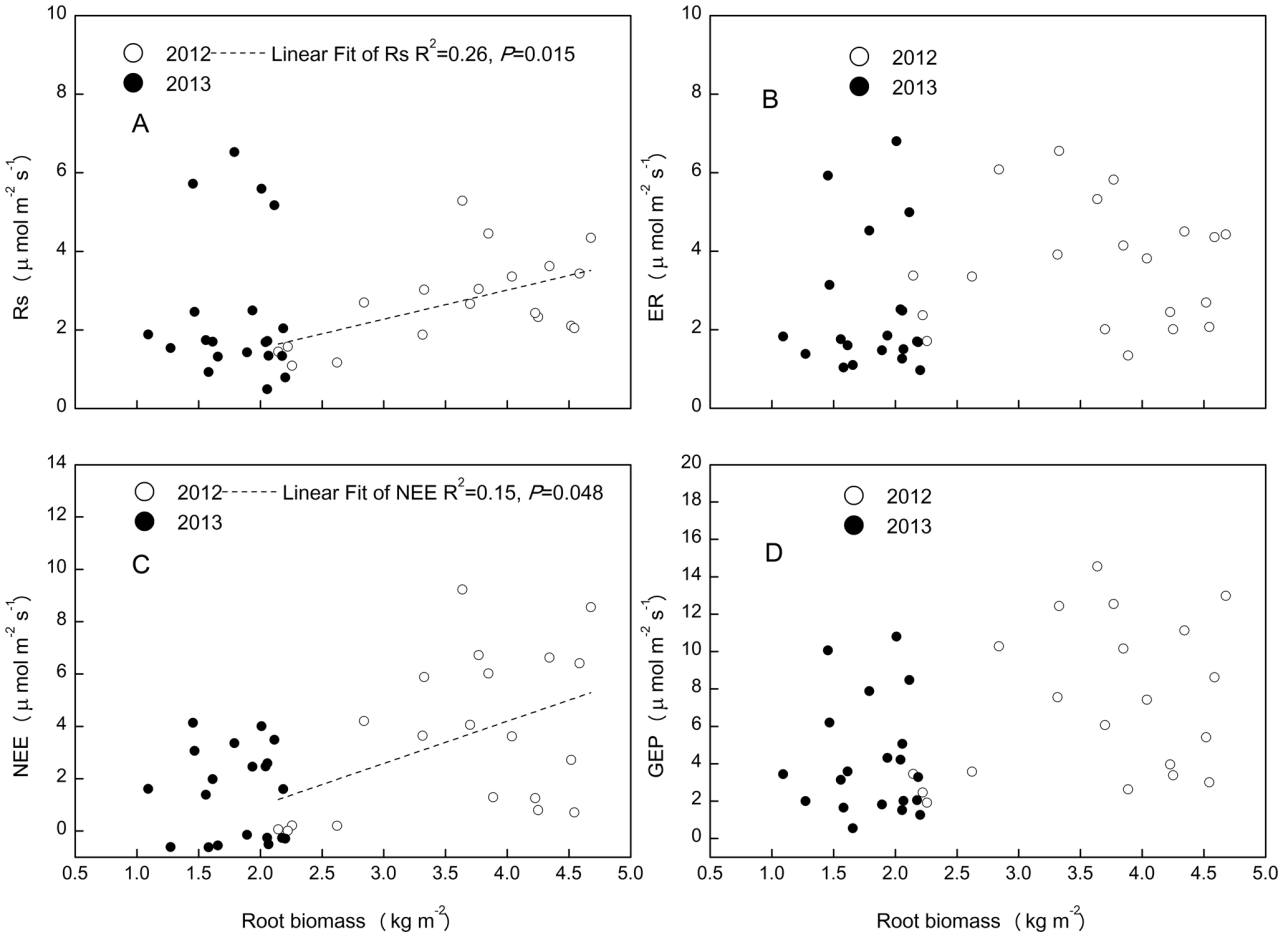
Relationship between root biomass and ecosystem C fluxes: soil respiration (Rs), ecosystem respiration (ER), net ecosystem exchange (NEE) and gross ecosystem productivity (GEP).

The statistical interaction term of above-ground biomass index and soil moisture showed polynominal relationship only with Rs in 2012 (Fig. 8a), whereas it linearly correlated with all the ecosystem C fluxes in 2013 (Fig. 8). The interaction term of above-ground biomass and soil temperature explained more variation in ecosystem C fluxes than did the interaction term of above-ground biomass and soil moisture (Figs. 8 and 9). The fitting slope between Rs and the interaction of above-ground biomass and soil temperature was higher in 2013 than in 2012 (Fig. 9a), but that between NEE and the interaction of above-ground biomass and soil temperature was smaller in 2013 than in 2012 (Fig. 9c). Root biomass only had effect on ecosystem C fluxes in wet year (Fig. 10).

## Discussion

### C fluxes and their inter-annual variation

In this alpine meadow ecosystem studied in the QTP, a higher uptake of C (GEP) than release (ER) resulted in a net C sink (Figs. 3–4). This result is similar to that reported in seasonally frozen areas in the QTP [23], [24], and in some arctic ecosystems [7].

Higher precipitation and associated higher soil moisture in 2012 than in 2013, and the similar MAT between the two years suggest that drought reduced annual ER, NEE and GEP in 2013. Our results are in agreement with those from temperate grassland ecosystems [37], [40], [41]. The quadratic relationship between ecosystem C fluxes and soil moisture (Table 3) at the temporal scale support the above findings, as ecosystem C fluxes were positively related to soil moisture in 2013 (Fig. 6). There was greater fluctuation in GEP than ER: GEP was 34% lower in 2013 than in 2012, compared to ER, which was 22% lower in 2013 than in 2012. These results are consistent with those from boreal and temperate forests [42] and temperate grasslands [40], which indicate that GEP is more sensitive to inter-annual climatic variation than ER in alpine meadow ecosystems. Differences in the magnitudes of the inter-annual variation in GEP and ER could be explained by differences in the slopes between these variables when plotted against soil moisture (Fig. 6). The greater dependence of GEP than ER on soil moisture across both years suggests that GEP is more sensitive to changes in soil moisture than ER. Despite the significant inter-annual difference in ER, NEE and GEP, Rs did not differ significantly between the two years. This indicates that annual variations in Rs may be controlled by other factors, such as soil temperature, as Rs had the highest slope when plotted against this variable (Fig. 7).

Drought typically reduces aboveground biomass in grasslands [43], [44]. However, in our study, no significant reduction in AGB was observed in 2013. One reason could be the various reactions of different species to drought [45], [46], and this compensation may hold community AGB constant. The 50.2% decrease in RB in 2013 could be attributed to the reduction in soil moisture because RB in alpine ecosystems is positively correlated with annual precipitation [47]. The relative reduction rate of RB in our study (50.2% reduction in RB and 2.1 m^3^ m^−3^ reduction in soil moisture) was higher than in a temperate grassland ecosystem (23% reduction in RB and 5.2 m^3^ m^−3^ reduction in soil moisture) [48]. The divergent responses of RB in the surface and deep soil layers [46], [49] could cancel each other out and therefore lead to a lower relative decrease in RB across the whole soil profile.

However, only one wet and dry year was included in this study. The wet year was followed directly by a dry year, which might lowers the effect of drought because soil drought might lag the meteorological drought.

### Main effects of warming

Experimental warming stimulated GEP more than ER (1.49 µ mol m^−2^s^−1^ vs. 0.79 µ mol m^−2^s^−1^), leading to an increase in NEE in the warming treatment of this alpine meadow ecosystem in a permafrost area of the QTP. Warming effects on ecosystem C exchange are likely modulated by soil water regimes [7], [37]. For example, Oberbauer (2007) reported that higher soil moisture in wet tundra limited increases in ER relative to increases in GEP under warming conditions, indicating the dependence of the warming effect on hydrological conditions. In the current alpine meadow ecosystem, differences in the responses of NEE to warming between 2012 and 2013 (Table 2) differed from results from a temperate steppe, in which NEE demonstrated no change under a warming treatment over two hydrologically contrasting years [40]. Soil moisture showed positive impacts on C fluxes in 2013 and negative impacts on fluxes in 2012 (Fig. 6). ABG and RB were positively correlated with ecosystem C fluxes (Figs. 8, 9). As there were no significant effects of warming on AGB or RB in either year (Tables 1, 2), the significant increase in NEE in warmed plots in 2012 could be attributed to the higher stimulation of GEP (2.09 µ mol m^−2^s^−1^) than ER (1.07 µ mol m^−2^s^−1^).

The insensitivity of NEE to warming in 2013 could be attributed to the effect of the soil moisture deficit on GEP and ER (Table 2, Fig. 6). The positive responses of GEP and ER to warming (Table 1) are consistent with those in a tundra ecosystem [7], but differ from those in a subalpine meadow ecosystem, where soil moisture stress induced by warming reduced ER [27], [40]. ER is composed of Rs and respiration of AGB. Therefore, the significant increase in ER in 2012 could be attributed mainly to the stimulation in Rs (Table 2), as AGB was insensitive to warming (Table 2). This indicates that the response of ER to warming was determined by Rs even though AGB respiration is the major component of ER in alpine meadow ecosystems [50]. Rs is composed of root respiration and microbial decomposition of soil organic matter [51]. There was no significant change in RB at a depth of 0–10 cm in the warming treatment (Tables 1, 2), which suggests that the response of ecosystem C emission to warming is determined by soil organic matter decomposition. The non-significant response of ER to warming in 2013 likely resulted from lower soil moisture (less than 15%, the optimal soil moisture for ecosystem C fluxes, Table 3), and the warming-induced reduction in soil moisture (Fig. 2). This is because the negative effects of drought and warming induced soil water stress on Rs and ER, which could override the positive effect of warming on these variables, which has been shown for a Montane meadow [52] and a subalpine meadow ecosystem in the QTP [27]. The marginal increase in GEP in 2013 (Table 2) likely resulted from a change in the species composition, which was observed in an open top chamber warming experiment nearby our study site, where coverage of grass and sedges decreased but that of forbs increased with warming [46]. The increased forbs biomass could ameliorate the negative impact of warming-induced soil moisture stress and the effect of lower AGB on GEP [40].

Although experimental warming tends to have a positive effect on plant productivity across ecosystems, experiments in grasslands indicate that clear increases in plant productivity in response to warming are relatively rare [53]. We did not detect a significant change in RB. We attributed this to the fact that we sampled the RB at a depth of 0–10 cm, which was constrained by a reduction in soil water, whereas the RB at a depth of 10–50 cm was significantly stimulated by warming. In contrast to the response of forbs, sedges and grass [28] may have cancelled each other out, leading to the non-significant change in AGB in this alpine meadow ecosystem. AGB and RB were positively correlated with ecosystem C fluxes (Figs. 8, 9). There was no significant change in AGB or RB with warming, whereas ecosystem C fluxes were significantly stimulated by warming (Tables 1, 2). It is possible that this resulted from the large seasonal variation in AGB and RB compared to the relatively smaller warming-induced changes in these biomass pools.

### Main effects of clipping

There was no significant effect of clipping on C fluxes, which contrasted with results from other studies where increases in GEP, ER, and NEE have been reported for a temperature steppe [54] and tallgrass prairie [13], and decreases in GEP, ER, and NEE have been reported for a Swiss grassland [55]. The negative impact of clipping on ecosystem C fluxes is attributed to the grass being cut in the middle of the growing season, which may reduce the green leaf area and thus C fluxes [55]. Positive effects of clipping on C fluxes may result primarily from improved light conditions with the removal of standing litter [54] and compensatory growth from clipping [56]. In the current study, we clipped the plants in late September once they had started to senesce, and this could be one reason for the non-significant effect of clipping on GEP, NEE and ER. In addition, soil temperature has been found to influence CO_2_ exchange in alpine meadow ecosystems [24], and we did not detect a significant effect of clipping on soil temperature (Fig. 2). Besides temperature, biomass also affects C fluxes in an alpine meadow ecosystem [24], as was observed in our study (Fig. 8). The significant decrease in AGB in 2013 under the clipping treatment with a non-significant change in C fluxes, suggests that soil temperature is the major factor controlling the response of ecosystem C fluxes to clipping.

## Conclusion

Ecosystem C fluxes responded positively to elevated temperature, with a higher relative increase in GEP than in ER, leading to a net C gain in this alpine meadow ecosystem. Clipping and its interaction with warming had no significant effect on ecosystem C fluxes because clipping did not significantly affect soil temperature. In addition, this study was conducted during two hydrologically contrasting years (wet in 2012 and dry in 2013), which provided a unique opportunity to understand how drought affects ecosystem C fluxes and their response to warming and clipping in an alpine meadow ecosystem. In the dry year, positive effects of warming on ecosystem C fluxes were cancelled by lower soil moisture. However, we caution that our study encompassed only a single wet and dry year, and thus our inferences of drought need to be supported by future research. Our findings will improve our understanding of the response of ecosystem C fluxes to the combined effects of climate change factors and human activities in an alpine meadow ecosystem in the permafrost region of the QTP.
